# Supplementary material for: Fecal Metaproteomics Reveals Reduced Gut Inflammation and Changed Microbial Metabolism Following Lifestyle-Induced Weight Loss
Source: Biomolecules. 2021 May 12;11(5):726. doi: 10.3390/biom11050726 (PMC8150863; doi:10.3390/biom11050726)
Supplement: Supplementary file 1 [file biomolecules-11-00726-s001.zip › Supplementary note - conversion.pdf]

Supplementary Note 1: Detailed description of the methods used for the taxonomic and functional analysis of the microbial communities

Metaproteome Analysis

Metaproteome analyzes were performed similarly to previously published [*1*]. In brief, 200 µg sample, 1 g silica beads, 200 µL 2 M sucrose solution and 350 µL phenol (10 g phenol dissolved in 1 mL aqua dest.) were shaken 2 times in a ball mill for 40 s at 30 Hz for cell lysis (ball mill MM400 Retsch, Düsseldorf, Germany). After centrifugation (10,000× *g*, RT, 10 min), the upper phenol phase was transferred into a new 2 mL reaction tube and was washed with 500 µL 1 M sucrose solution in the ball mill for 5 min at 1800 rpm. After a further centrifugation step (10,000× *g*, RT, 10 min) the phenol phase was transferred again into a new 2 mL reaction tube and the proteins were precipitated over night at −20 °C by addition of 2 mL of ice-cold 100 mM ammonium acetate in methanol. The centrifugation and precipitation step was repeated once with 1.5 mL of ice-cold 100 mM ammonium acetate in methanol. The supernatant was removed. Subsequently, the pellet was dried and dissolved in 250 µL 7 M urea, 2 M thiourea (urea buffer) as well as 0.01 g/mL dithiothreitol. For better solubilization of the pellet, silica beads were added and the reaction tube was shaken in a ball mill for 3 minutes. After final centrifugation (10,000× *g*, 4 °C, 10 min), the supernatant was transferred into a new reaction tube and stored at −20 °C until further usage.

The amido black assay was used for protein quantification [*2,3*]. Therefore, 50 µL of the sample was precipitated with 300 µL amido black staining solution (0.26 mg/L amido black, 90% methanol, 10% acetic acid). The sample was centrifuged (16,400× *g*, RT, 5 min) and the supernatant was decanted. The pellet was washed twice to remove unbound dye with 500 µL 10% acetic acid in methanol. The supernatant was decanted after a further centrifugation step (16,400× *g*, RT, 5 min). Subsequently, the protein pellet was dissolved in 1 mL 100 mM sodium hydroxide solution and the absorption was measured in a photometer (Spectrophotometer Genesys 10S UV-Vis, Thermo Scientific, Waltham, USA) at wavelength 615 nm. The protein concentrations were calculated using a calibration curve generated with defined amounts of bovine serum albumin.

A slightly modified version of the FASP protocol [*4*] was used for tryptic digestion. First, 100 µg protein extract was loaded on a FASP filter (Pall Nanosep, MWCO 10 kDa Omega, VWR, Dresden) and the urea buffer was removed similar to all following buffers by centrifugation (10,000× *g*, RT, 20 min). Proteins were washed with 200 µL 8 M urea. The reduction of the disulfide bonds was achieved by adding 100 µL 40 mM dithiothreitol for 20 min at 56 °C on a thermomixer at 300 rpm followed by alkylation with 100 µL 55 mM iodoacetamidefor 20 min in the dark. Subsequently, one washing step with 100 µL 8 M urea and three washing steps with 100 µL 50 mM ammonium bicarbonate were carried out. The entire flow-through was discarded. For trypsinization, 200 µL 50 mM ammonium bicarbonate containing 1 µg trypsin was added and the samples were shaken for 2 h at 37 °C on a thermomixer. After a further centrifugation step (10,000 G, RT, 5 min), remaining peptides on the filter were rinsed by adding 50 µL extraction buffer (50 mM ammonium bicarbonate, 5 % acetonitrile) and 50 µl LC-MS grade water. Finally, the filters were removed and the samples were stored at −20 °C. Before LC-MS/MS analysis, 30 µL peptide solution were acidified with 3 µL 0.5% TFA and transferred into a vial.

The samples were analysed using a LC-MS/MS system comprising an UltiMate 3000 RSLC nano splitless liquid chromatography system, coupled online to an Orbitrap Elite™ Hybrid Ion Trap-Orbitrap MS (both from Thermo Fisher Scientific, Bremen, Germany). After injection, peptides were loaded isocratically on a trap column (Dionex Acclaim, nano trap column, 100 µm i.d. × 2 cm, PepMap100 C18, 5 µm, 100 Å, nanoViper) with a flow rate of 7 µL/min chromatographic liquid phase A (98 % LC-MS Water, 2 % ACN, 0.05 % TFA) for desalting and concentration. Chromatographic separation was performed on a Dionex Acclaim PepMap C18 RSLC nano reversed-phase column (2 µm particle size,

100 Å pore size, 75 µm inner diameter and 250 mm length) at 40 °C column temperature. A flow rate of 400 nL/min was applied using a binary A/B-solvent gradient (solvent A: 98 % LC-MS Water, 2% acetonitrile, 0.1% formic acid; solvent B: 80 % acetonitrile, 10% LC-MS Water, 10 % trifluoroethanol, 0.1 % formic acid) starting with 4 % solvent B for 4 min, continuing with a linear increase to 55% solvent B within 120 min, followed by a column wash with 90% solvent B for 5 min and a re-adjustment with 4% solvent B for 25 min. For MS acquisition, a data-dependent MS/MS method was chosen. For the conducted measurements, MS was operated in positive ion mode, and precursor ions were acquired in the orbital trap of the hybrid MS at a resolution of 30,000 and an m/z range of 350–2000. Subsequently, the fragment ion scan has proceeded in the linear ion trap of the hybrid MS with a mass range and a scan rate with "normal" parameter settings for the top 20 most intense precursors selected for collision-induced dissociation.

The protein identification was carried out with the MetaProteomeAnalyzer (version 3.0) [*1*] and the search engines X!Tandem [*5*], OMSSA [*6*] and Mascot [*7*] were used with the following parameters: enzyme trypsin, one missed cleavage, monoisotopic mass, carbamidomethylation (cysteine) as fixed modification, oxidation (methionine) as variable modifications, ± 10ppm precursor and ± 0.5 Da MS/MS fragment tolerance, 1^{13}C, +2/+3 charged peptide ions and a false discovery rate of 1%. The protein database comprised the UniProtKB/SwissProt database (date 16/01/2019) and the metagenome published by Qin et al. [*8*]. Access to the MS files is granted by the PRIDE database [*9*] with the accession number PXD020902. For identified proteins lacking a taxonomic or functional annotation BLAST search was conducted (NCBI-Blast-version 2.2.31 [*10*]) against UniProtKB/SwissProt. All BLAST hits with e-values below 10^{-4} were merged and used for protein identification. Redundant homologeous protein identifications were merged to a protein group (metaprotein) if they had at least one peptide identification in common.

Finally, a matrix of all metaproteins across all samples was exported, which consisted of the meta information provided by the UniProtKB database [*11*]: NCBI taxonomy, enzyme commission numbers (EC), KEGG orthologies (KO), UniProtKB reference clusters and UniProtKB keywords.

References

1. Heyer, R.; Schallert, K.; Büdel, A.; Zoun, R.; Dorl, S.; Behne, A.; Kohrs, F.; Püttker, S.; Siewert, C.; Muth, T.; et al. A Robust and Universal Metaproteomics Workflow for Research Studies and Routine Diagnostics Within 24 h Using Phenol Extraction, FASP Digest, and the MetaProteomeAnalyzer. *Front. Microbiol.* **2019**, *10*, 1883, doi:10.3389/fmicb.2019.01883.
2. Racusen, D. Stoichiometry of the amido black reaction with proteins. *Anal. Biochem.* **1973**, *52*, 96–101, doi:10.1016/0003-2697(73)90334-5.
3. Popov, N., Schmitt, M., Schulzeck, S., Matthies, H. Eine störungsfreie Mikromethode zur Bestimmung des Proteingehaltes in Gewebehomogenaten. *Acta Biol Med Ger* 1975, *34*, 1441–6, doi:.
4. Wiśniewski, J.R., Zougman, A., Nagaraj, N., Mann, M. Universal sample preparation method for proteome analysis. *Nat Methods* **2009**, *6*, 359–62, doi:10.1038/nmeth.1322.
5. Craig, R.; Beavis, R.C. TANDEM: matching proteins with tandem mass spectra. *Bioinform.* **2004**, *20*, 1466–1467, doi:10.1093/bioinformatics/bth092.
6. Geer, L.Y.; Markey, S.P.; Kowalak, J.A.; Wagner, L.; Xu, M.; Maynard, D.M.; Yang, X.; Shi, W.; Bryant, S.H. Open Mass Spectrometry Search Algorithm. *J. Proteome Res.* **2004**, *3*, 958–964, doi:10.1021/pr0499491.
7. Perkins, D.N., Pappin, D.J., Creasy, D.M., Cottrell, J.S. Probability-based protein identification by searching sequence databases using mass spectrometry data. *Electrophoresis* **1999**, *20*, 3551–67, doi:10.1002/(SICI)1522-2683(19991201)20:18<3551::AID-ELPS3551>3.0.CO;2-2.
8. Qin, J.; Li, R.; Raes, J.; Arumugam, M.; Burgdorf, K.S.; Manichanh, C.; Nielsen, T.; Pons, N.; Levenez, F.; Yamada, T.; et al. A human gut microbial gene catalogue established by metagenomic sequencing. *Nature* **2010**, *464*, 59–65, doi:10.1038/nature08821.
9. Vizcaíno, J.A., Csordas, A., del-Toro, N., Dianes, J.A., Griss, J., Lavidas, I., Mayer, G.; Perez-Riverol, Y.; Reisinger, F.; Ternent, T. et al. 2016 update of the PRIDE database and its related tools. *Nucleic Acids Res,* **2016**, *44*, 56, doi:10.1093/nar/gkv1145.
10. Camacho, C.; Coulouris, G.; Avagyan, V.; Ma, N.; Papadopoulos, J.S.; Bealer, K.; Madden, T.L. BLAST+: architecture and applications. *BMC Bioinform.* **2009**, *10*, 421, doi:10.1186/1471-2105-10-421.
11. Patient, S.; Wieser, D.; Kleen, M.; Kretschmann, E.; Martin, M.J.; Apweiler, R. UniProtJAPI: a remote API for accessing UniProt data. *Bioinform.* **2008**, *24*, 1321–1322, doi:10.1093/bioinformatics/btn122.
